# Psychological and Physiological Assessment of Distress Among Public Healthcare Workers During Pandemic Control Efforts

**DOI:** 10.3390/healthcare14020212

**Published:** 2026-01-14

**Authors:** Dinko Martinovic, Anamarija Jurcev Savicevic, Majda Gotovac, Zeljko Kljucevic, Magda Pletikosa Pavic, Marko Kumric, Zeljka Karin, Slavica Kozina, Daniela Supe Domic, Manuel Colome-Hidalgo, Josko Bozic

**Affiliations:** 1 Department of Maxillofacial Surgery, University Hospital of Split, 21000 Split, Croatia; 2 Department of Maxillofacial Surgery, School of Medicine, University of Split, 21000 Split, Croatia; 3 Department of Preventive Medicine, Faculty of Health Sciences, University of Split, 21000 Split, Croatia; 4 Teaching Public Health Institute of Split-Dalmatia County, 21000 Split, Croatia; 5 Department of Public Health, School of Medicine, University of Split, 21000 Split, Croatia; 6 Department of Pathophysiology, School of Medicine, University of Split, 21000 Split, Croatia; 7 Laboratory for Cardiometabolic Research, School of Medicine, University of Split, 21000 Split, Croatia; 8 Department of Psychological Medicine, School of Medicine, University of Split, 21000 Split, Croatia; 9 Department of Medical Laboratory Diagnostics, Faculty of Health Sciences, University of Split, 21000 Split, Croatia; 10 Department of Medical Laboratory Diagnostics, University Hospital of Split, 21000 Split, Croatia; 11 Area de Ciencias de la Salud, Instituto Tecnologico de Santo Domingo (INTEC), Santo Domingo 10602, Dominican Republic; 12 Instituto de Investigacion en Salud, University Autonoma de Santo Domingo (UASD), Santo Domingo 10602, Dominican Republic

**Keywords:** public health workers, pandemic, crisis situations

## Abstract

**Background/Objectives:** Public healthcare workers face significant occupational stress during crisis situations, yet research on this particular population remains limited compared to other healthcare workers. The aim of this study was to investigate the impact of the COVID-19 pandemic on distress levels and the sense of coherence among public health workers by integrating psychological assessments with physiological markers of stress to identify protective factors against pandemic-related occupational stress. **Methods:** This longitudinal study was conducted at the Teaching Public Health Institute of Split and Dalmatia County from July 2021 to February 2022 at two time points: the latency phase (between COVID-19 waves) and hyperarousal phase (during an active wave). Fifty-four public health workers participated in the study. There were three questionnaires assessing psychological distress: Kessler Psychological Distress Scale, Impact of Events Scale—Revised and Sense of Coherence Scale-29. Salivary and blood samples were collected at both time points to measure cortisol levels, cortisol awakening response, and interleukin-6 concentrations. **Results:** The cortisol area under the curve with respect to ground (AUCg) was significantly elevated during the stress phase compared to the latency phase (234.8 vs. 201.8; *p* = 0.023), indicating heightened physiological stress responses. Epidemiologists demonstrated significantly lower sense of coherence scores compared to non-epidemiologists (117.9 ± 9.1 vs. 125.6 ± 10.5; *p* = 0.029). A lower sense of coherence was significantly associated with higher psychological distress and post-traumatic stress symptoms. Multiple linear regression analysis revealed that sense of coherence and interleukin-6 levels were significant independent predictors of cortisol changes. **Conclusions:** The findings demonstrate that public health workers experience measurable physiological stress responses during pandemic peaks, with sense of coherence emerging as a protective psychological factor. Interventions targeting sense of coherence and organizational support may possibly enhance resilience and reduce mental health morbidity in this vulnerable workforce during crisis situations.

## 1. Introduction

Healthcare workers (HCWs), particularly physicians, have long been recognized as a population exposed to elevated levels of occupational distress [1]. Even prior to the emergence of the COVID-19 pandemic, physicians were already identified as a group experiencing significant psychological strain compared to the general population, with persistent workplace tension often culminating in exhaustion and psychological distress [2,3]. The advent of the COVID-19 pandemic intensified these challenges, as HCWs worldwide faced unprecedented demands and heightened stress [4]. The healthcare system’s primary focus shifted to preventing the transmission of SARS-CoV-2 and treating those afflicted with COVID-19, thereby subjecting HCWs to new and intensified stressors. These included adapting to stringent infection control protocols, coping with shortages of protective equipment, witnessing high patient mortality, and managing the increased personal risk of infection, as well as concern for colleagues, family, and friends. Despite this evident psychological burden, insufficient attention has been directed toward the critical issue of occupational burnout and the overall psychological well-being of HCWs. Although the burden of the pandemic was broadly shouldered by HCWs, they are often not recognized as a vulnerable group, resulting in limited focus on their mental health and morbidity. There is a growing concern regarding the long-term mental and physical health consequences for HCWs, particularly after enduring two years of pandemic-related occupational and personal stressors, restrictions in private and family life, and the absence of substantial governmental interventions to mitigate COVID-19-specific risk factors [5].

Research on the mental health of HCWs during the pandemic has predominantly centered on those directly involved in patient care, especially within hospital settings [6]. However, only a limited number of studies have examined potential differences between frontline and non-frontline HCWs, and even fewer have investigated the mental health of public health workers (PHWs) [7]. PHWs, despite their indispensable role in pandemic control, typically do not encounter the same level of acute stress as clinicians or intensive care physicians in their routine work. Nevertheless, during the COVID-19 pandemic, PHWs were confronted with extreme workloads and stressors for which they were neither trained nor accustomed [8]. To date, psychological distress and protective behaviors among PHWs in the context of COVID-19 have not been systematically assessed. Within the Croatian healthcare system, public health is a vital sector, with PHWs responsible for safeguarding community health through a range of interventions. PHWs, which include epidemiologists, microbiologists, sanitary engineers, nurses, and other health professionals, operate across diverse settings such as state and local health departments, hospitals, universities, laboratories, and schools. Their primary responsibilities encompass the detection, prevention, and control of infectious diseases, the implementation of immunization programs, and conducting epidemiological surveys. Routine duties also include epidemiological investigations, enforcement of isolation measures, environmental disinfection, vector control, and specimen collection and analysis within the community.

The literature indicates that the most prevalent mental health conditions associated with the pandemic among HCWs are depression, stress, and burnout, often linked to factors such as fear of infection, inadequate protective equipment, increased job demands, and insufficient family support [9,10]. Most studies have focused on identifying risk factors affecting HCWs during the pandemic, with relatively few exploring protective factors that may buffer against stress [11]. A recent rapid systematic review by Hannemann et al. identified 46 studies examining the impact of the COVID-19 pandemic on the mental health of HCWs [12]. The review concluded that HCWs face multiple pandemic-related stressors, including increased workload, fear of infecting themselves or others, concerns about family and friends, loss-related events, and general distress. Psychosocial resources such as resilience, active and emotion-focused coping strategies, and social support were found to be statistically associated with reduced mental health problems among HCWs. Furthermore, organizational factors such as clear communication, colleague support, and adequate organizational backing possibly play a significant role in influencing psychosocial outcomes.

In addition to psychological assessments, the measurement of biological markers has emerged as a valuable approach to quantifying stress responses [13]. The glucocorticoid response, particularly the diurnal rhythm of cortisol secretion, serves as a reliable biomarker of stress and emotional well-being [14]. Cortisol exhibits a pronounced diurnal pattern, peaking shortly after awakening (the cortisol awakening response, CAR) and gradually declining throughout the day to reach its lowest levels in the evening. The total diurnal cortisol output, often estimated as the area under the curve with respect to ground (AUCg), along with the CAR, is a widely utilized index of diurnal cortisol functioning and provides objective measures of stress response [15]. In HCWs, especially those exposed to high work demands, shift work, and psychosocial stressors, alterations in CAR and AUCg have been documented as a physiological correlate of occupational stress and burnout [16,17]. Recent studies have provided empirical evidence linking CAR and AUCg to stress and burnout among HCWs. For instance, a study on female nurses working different shifts demonstrated that shift work itself is a significant stressor, with notable differences in CAR indices between night-shift and day-shift nurses [17]. Nurses working night shifts exhibited altered CAR patterns, reflecting the physiological impact of disrupted sleep–wake cycles and heightened occupational stress. Moreover, a study by Morera et al. compared AUCg values among four different worker profiles: engaged, strained, cynical, and burned-out [18]. The findings demonstrated that the “strained” group, characterized by high dedication but also high exhaustion, exhibited significantly higher AUCg values than the engaged group, reflecting increased cortisol output in response to heightened job demands. Furthermore, several studies have shown that HCWs with high levels of burnout exhibit elevated concentrations of salivary cortisol, particularly in the morning and afternoon, suggesting an association between increased CAR and burnout symptoms [19,20]. However, the relationship between CAR and burnout is complex and not entirely uniform across studies. A meta-analysis by Chida and Steptoe found that CAR was negatively related to fatigue, burnout, or exhaustion, yet individual studies have reported both elevated and non-elevated awakening cortisol levels in subjects with clinical burnout [21]. Some research suggests that individuals with severe burnout may exhibit a hypoactive HPA axis, while others show normal or even hyperactive responses.

Henceforth, the primary aim of this study is to investigate the impact of the COVID-19 pandemic on anticipated distress levels and the sense of coherence among PHWs engaged in pandemic control efforts. By integrating psychological assessments with biological markers of stress, this study seeks to provide a comprehensive understanding of the mental health challenges faced by PHWs and to identify potential protective factors that may mitigate the adverse effects of pandemic-related occupational stress.

## 2. Materials and Methods

### 2.1. Study Design and Ethical Considerations

This longitudinal study was conducted at the Teaching Public Health Institute of Split and Dalmatia County in the time period from July 2021 to February 2022. The study was performed at two time points, the first between two waves of the COVID-19 pandemic in Croatia (latency phase), from 1 July 2021 to 31 October 2021, and the second time point during the wave of the COVID-19 pandemic (hyperarousal phase), from 1 November 2021 to 28 February 2022.

The study was conducted according to the guidelines of the Declaration of Helsinki and approved by the Ethics Committee of the Teaching Public Health Institute of Split and Dalmatia County (Class:500-01/21-01/20; No. 2181-103-01-21-1; Date: 8 July 2021). Prior to study inclusion, all of the participants were informed about the purpose and aims of this study and they signed written consent.

### 2.2. Participants

The study was performed on healthcare workers at the Teaching Public Health Institute of Split and Dalmatia County. Participation was anonymous and voluntary, without any compensation. All of the subjects were recruited using official e-mail addresses and mobile phone messages. The inclusion criteria were healthcare workers and involvement in COVID-19 pandemic control work.

The exclusion criteria were self-reported preexisting mental disorders that require psychopharmacological treatment; hypothalamic–pituitary–adrenal axis disorders; pregnancy and/or breastfeeding; use of corticosteroid medications; and oral contraceptives and hormone replacement therapy. There were 62 healthcare workers eligible for inclusion, out of which 54 were included in the study. Henceforth, the proportion ratio was 87.7% and the sample consisted of 17 medical doctors, 16 sanitarians, 18 medical laboratory technicians, and 3 public health nurses. Regarding the task distribution, 33 of them worked in the Department of Epidemiology due to COVID-19 contact tracing, quarantine management and COVID-19 vaccination, and 21 of them worked in the Department of Microbiology, collecting and testing upper respiratory specimens for current SARS-CoV-2 infection. Hence, we have included almost all of the PHWs from the Teaching Public Health Institute of Split and Dalmatia County.

### 2.3. Questionnaires and Study Variables

The questionnaires were administrated using the online survey created through the Google Forms^®^ platform (Google LLC, Mountain View, CA, USA). The link to the survey was distributed through e-mail addresses and mobile phone messages. The survey consisted of 4 questionnaires and it was completed only once during the first phase of the study (latency phase).

The first part of the survey was a semi-structured questionnaire regarding the participant’s sociodemographic information such as age, gender, marital status, smoking, education level, occupation, employment status, working experience, working experience with COVID-19, and COVID-19 vaccination.

The second questionnaire was the Kessler Psychological Distress Scale (K10) [22]. It is a widely validated structured screening instrument designed to measure non-specific psychological distress. It contains 10 items that assess how often during the past 4 weeks respondents experienced symptoms of distress. It is self-reported and each item is graded on a 5-point Likert scale with a total score range from 10 to 50. Higher scores indicate greater psychological stress.

The third questionnaire was the Impact of Events Scale—Revised (IES-R) [23]. It is a validated self-reported structured measure for assessing post-traumatic stress symptoms following traumatic events. It consists of 22 items which evaluate three symptoms clusters: intrusion, avoidance and hyperarousal. The symptom frequency over the past 7 days is graded on a 5-point Likert scale, with the total score ranging from 0 to 88. Higher scores indicate a greater potential post-traumatic stress concern.

The fourth questionnaire was the Sense of Coherence Scale (SOC-29) [24]. It is a structured, validated psychometrically robust instrument designed to measure an individual’s capacity to perceive life as comprehensible, manageable, and meaningful. It consists of 29 items which evaluate three subscales: comprehensibility (11 items), manageability (10 items), and meaningfulness (8 items). Each item is scored on a 7-point Likert scale with the total score range from 29 to 203. Higher scores indicate a stronger sense of coherence.

### 2.4. Laboratory Analysis

All biological materials—saliva and blood—were collected at both time points (latency phase and hyperarousal phase). Furthermore, all of the procedures and analyses were conducted according to international standards in the same laboratory and by the same experienced medical biochemist who was blinded to the subject group in the study.

Saliva samples for the determination of salivary cortisol were obtained using the Salivette commercial kit^®^ (Sarstedt Inc., Nombrecht, Germany) and collected according to the protocol for unstimulated, passive saliva sampling. Participants were given written and oral instructions on how to adequately collect 4 saliva samples across the day, according to the given protocol. Due to the circadian rhythm of secretion, saliva samples were collected on the same day in the exact same order: immediately after awakening, 30 min after awakening, 60 min after awakening, and just before going to sleep. They were informed that at least half an hour before sampling, they should not eat; drink coffee, alcohol, or juice; smoke; or exercise to avoid a stressful mood. They were also told to rinse their mouths with water before giving the sample and not to brush their teeth or chew gum. Participants collected saliva samples at home, recorded each sampling time and stored samples in a home refrigerator at +2 °C to +8 °C until they were admitted by the researcher (up to 2 days at the latest). The Salivette devices were delivered to the Department of Medical Laboratory Diagnostics, University Hospital of Split, Croatia, and stored at −20 °C until analysis. When the analyses were conducted, the frozen samples were first kept at the room temperature for 60 min and then centrifuged at 1500× *g* for three minutes. Salivary cortisol concentration was analyzed by the immunochemical CLIA method on the IDS-iSYS Multi-Discipline Automated System. The lower sensitivity limit of salivary cortisol concentration according to the manufacturer is 0.02 µg/dL. The linearity range is from 0.02 to 3.00 µg/dL.

Blood samples were collected after overnight fasting, no later than 08:00 in the morning on the day after saliva sampling. Using a sterile disposable needle, venous blood was obtained from the cubital vein into a vial with clot activator (BD Vacutainer^®^ CAT-Clot Activator Tube, 2.0 mL, Becton, Dickinson and Company, Franklin Lakes, NJ, USA). Samples were centrifuged and part of the sampled blood was immediately analyzed (cortisol), whereas part of the sample was aliquoted and stored at −80 °C for analysis of IL-6. Both serum cortisol and IL-6 were analyzed using the electrochemiluminescence immunoassay “ECLIA” on Cobas immunoassay analyzers (Cobas e801 Roche Diagnostics GmbH, Mannheim, Germany) according to the manufacturer’s instructions.

### 2.5. Statistical Analysis

Analyses of the data were conducted using computer software MedCalc (MedCalc Software, Ostend, Belgium, version 23.2.0). The normality of data distribution was estimated using the Kolmogorov–Smirnov test. All quantitative variables were presented as mean ± standard deviation or median and interquartile range, depending on their normality of distribution. All qualitative variables were presented as whole numbers and percentages. Depending on the normality of distribution, Student *t*-tests and Mann–Whitney U tests were used for statistical comparison of quantitative variables between two groups, while the Chi-squared test was used for comparison of qualitative variables. However, for comparison of the same quantitative variables between two different time points, paired *t*-tests or Wilcoxon tests were used, depending on the normality of distribution. Spearman’s rank correlation coefficient was used to test association between non-parametric variables. Additionally, multiple linear regression analysis was used to determine significant predictors for the Δ cortisol levels as a dependent variable. The independent variables in this analysis were SOC-29 score, K10 score, IES-R score, Δ IL-6, age and working experience. From these analysis, we reported the *p*-values with unstandardized β-coefficients, standard error and t-values. The level of statistical significance was set at *p*-value < 0.05.

## 3. Results

This study included 54 participants with a median age of 45.5 (29.0–54.0) years. There were 11 (20.4%) males and 43 (79.6%) females and most participants were married (64.8%) and had a master’s degree education (55.6%). Furthermore, most of the participants had working experience of >20 years (37.0%) and has been working with COVID-19 for more than 12 months (64.8%). Moreover, most participants were vaccinated for COVID-19 (53.7); of these, 7 (13.0%) had received one dose and 22 (40.7%) has received two doses (Table 1).

In the comparison between non-epidemiologists and epidemiologists, it was found that the latter were significantly older (*p* = 0.003), had a higher education level (*p* = 0.004), had a longer working experience (*p* = 0.016), and had a longer working experience with COVID-19 (*p* = 0.012). Moreover, significantly more of them had previously contracted COVID-19 infection (*p* = 0.042) (Table 1).

Regarding the questionnaire score, the epidemiologists had a statistically lower SOC-29 score compared to the non-epidemiologists (117.9 ± 9.1 vs. 125.6 ± 10.5; *p* = 0.029) (Table 2). There were no other significant differences between the two groups.

**Figure 1:**
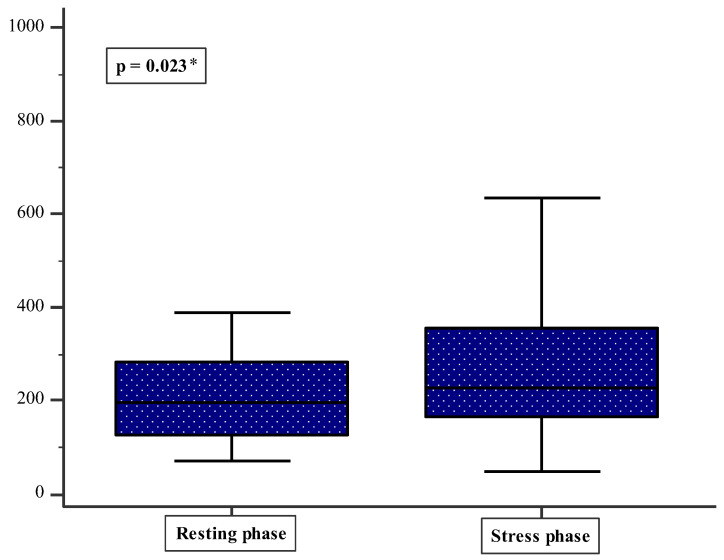
Comparison of cortisol AUCg between the resting phase and the stress phase. * Wilcoxon test.

After comparing the laboratory analysis results between the resting phase and the stress phase, a statistically significant difference was found in cortisol AUCg (Figure 1). It was found that the cortisol AUCg is significantly higher in the stress phase compared to the resting phase (201.8 (126.0–286.6) vs. 234.8 (164.3–351.3); *p* = 0.023) (Figure 1). There were no other significant laboratory differences in the cortisol and IL-6 levels between the resting phase and the stress phase (Table 3).

The correlation analysis showed that there is a significant medium negative correlation between the SOC-29 score and K10 score (r = −0.428, *p* < 0.05) and a significant weak negative correlation between SOC-29 score and IES-R score (r =−0.277, *p* < 0.05) (Table 4). Furthermore, there was a significant medium positive correlation between IES-R score and K10 score (r = 0.536, *p* < 0.05).

Multiple linear regression analysis showed that the SOC-29 score (β ± SE, −2.425 ± 1.164, *p* = 0.042) and Δ IL-6 levels (12.678 ± 6.276, *p* = 0.049) significantly predicted the Δ cortisol levels after model adjustment for age and working experience, with K10 and IES-R scores as dependent variables (Table 5). The overall regression model was statistically significant (*p* = 0.048) with a coefficient of determination (R2) of 0.219 (R2 adjusted = 0.120).

## 4. Discussion

The main findings of this longitudinal study revealed that cortisol AUCg was significantly elevated during the pandemic’s hyperarousal (stress) phase compared to the latency (resting) phase. Furthermore, the results showed that epidemiologists demonstrated significantly lower SOC-29 scores compared to non-epidemiologists, suggesting diminished psychological resilience in this group. Also, a lower sense of coherence was associated with higher psychological distress and post-traumatic stress symptoms, while both a sense of coherence and IL-6 levels emerged as significant independent predictors of cortisol responses after controlling for age and work experience. These findings provide novel insights into the occupational stressors experienced by PHWs, a population that has received limited attention compared to frontline clinical healthcare workers during the pandemic.

The significant elevation in cortisol AUCg during the pandemic’s active phase compared to the resting phase demonstrates a measurably higher stress response among PHWs. These findings align with emerging evidence documenting altered HPA axis functioning in HCWs during COVID-19 [25]. Ibar et al. reported that 40% of HCWs at a hospital in Argentina exhibited altered hair cortisol levels during the pandemic’s first wave, establishing cortisol as a viable biomarker of chronic stress in this context [14]. Likewise, Rajcani et al. found that more than 75% of HCWs agreed that COVID-19 led to increased workplace stress along with corresponding elevations in hair cortisol concentrations. AUCg represents total cortisol output throughout the day and serves as a comprehensive measure of HPA axis activity [26]. The aforementioned research has demonstrated that AUCg is sensitive to occupational stress and burnout in healthcare populations. Fekedulegn et al. emphasized that AUCg provides critical information about the magnitude of cortisol response, distinct from pattern-based measures such as the cortisol awakening response [27]. Hence, the elevated AUCg observed in this study during the hyperarousal phase suggests increased allostatic load, potentially placing PHWs at risk for long-term health consequences, including cardiovascular disease, metabolic dysregulation, and immune dysfunction.

Similar to this study, Fortuna et al. conducted an analysis during the second wave of COVID-19 in Argentina and found that 10% of HCWs exhibited hair cortisol levels above the reference threshold, with associations between high cortisol levels and age, workload, emotional exhaustion and depersonalization [14]. The variation in cortisol levels across different pandemic phases, as observed in both the Fortuna et al. study and the present study, suggests that the intensity and duration of pandemic-related stressors influence HPA axis activation. The relationship between cortisol and burnout is complex and not entirely consistent across studies [28,29,30]. While some investigations have reported elevated cortisol levels in HCWs with high burnout symptoms, others have documented blunted or hypoactive HPA axis responses in individuals with severe burnout. McCanlies et al. found that higher burnout symptoms were associated with higher cortisol levels in some studies of medical professionals, including elevated diurnal salivary cortisol [31]. However, their own research in police officers revealed that exhaustion and depersonalization were negatively associated with the cortisol AUCg, which suggests a downregulation of the HPA axis in response to chronic occupational stress. This apparent contradiction may reflect different stages of stress adaptation with initial hyperactivation of the HPA axis transitioning to hypocortisolism as burnout becomes more severe and chronic.

Another interesting finding is that epidemiologists exhibited significantly lower SOC-29 scores compared to non-epidemiologists. Sense of coherence, conceptualized by Antonovsky, encompasses three dimensions: comprehensibility, manageability and meaningfulness. Higher SOC-29 scores have been consistently associated with better mental health outcomes, lower stress, and enhanced resilience across diverse populations [32,33]. The lower SOC among epidemiologists may reflect the specific occupational demands imposed by pandemic control activities. Epidemiologists in this study were significantly older, more experienced and had worked longer on COVID-19-related tasks compared to non-epidemiologists. They were responsible for contact tracing, quarantine management, epidemiological investigations, and vaccination coordination. All of these activities are characterized by high uncertainty, rapidly changing guidelines, extensive workload, and direct exposure to public distress. These responsibilities may have eroded their sense of comprehensibility due to unpredictable disease patterns and policy changes, their manageability due to resource limitations and overwhelming caseloads, and their meaningfulness due to burnout and loss of professional fulfillment. Stoyanova et al. examined sense of coherence and burnout in Bulgarian HCWs during the pandemic and found that all components of SOC were positively correlated with personal accomplishment, while emotional exhaustion and depersonalization correlated negatively with SOC [34]. Their regression analyses revealed that SOC components explained 31% to 44% of the variance in burnout dimensions, confirming SOC’s moderating role in occupational stress. González-Siles et al. similarly reported in their systematic review that HCWs with high SOC scores experienced greater personal fulfillment, while a low SOC was related to higher burnout levels [35]. These findings are consistent with the present study’s observation that a lower SOC was associated with higher psychological distress (K10) and post-traumatic stress symptoms (IES-R). Furthermore, these associations align with the salutogenic model’s predictions. Basson and Rothmann found that SOC was significantly related to emotional exhaustion, depersonalization, and personal accomplishment in pharmacists [36]. Moreover, Paterson et al. demonstrated that sense of coherence was a significant protective factor for mental well-being, with increased coherence associated with reduced symptoms of anxiety, depression, and burnout [37]. The protective role of SOC against stress is particularly relevant for HCWs who often face chronic occupational stressors rather than acute traumatic events. The ability to perceive one’s work environment as comprehensible, manageable, and meaningful may buffer against the psychological toll of sustained pandemic response efforts. Interventions designed to strengthen SOC components, such as providing clear communication, ensuring adequate resources, fostering organizational support, and emphasizing the meaningfulness of public health work, may enhance resilience and reduce mental health morbidity in this population.

The finding that IL-6 levels independently predicted cortisol responses is an interesting contribution regarding psychoneuroimmunology and occupational health. IL-6 is a pro-inflammatory cytokine that plays a central role in the acute phase response and also functions as a marker of chronic stress and inflammation in non-infected individuals [38]. Amer et al. investigated the relationship between IL-6 levels and stress, anxiety, and depressive symptoms among healthcare workers and found associations between elevated IL-6 and mental health outcomes [39]. The mechanistic pathway linking stress, IL-6, and cortisol is bidirectional: psychological stress activates the HPA axis, leading to cortisol secretion, which in turn modulates immune function and cytokine production. Conversely, inflammatory cytokines such as IL-6 can stimulate the HPA axis, creating a feedback loop that perpetuates physiological stress responses. The independent predictive value of IL-6 for cortisol levels observed in this study suggests that inflammatory processes contribute to HPA axis dysregulation beyond the effects of psychological factors alone.

This study had several limitations. While the relatively small sample size was adequate for the longitudinal design and biological assessments, on the other hand, it limited the statistical power and generalizability of the findings. Furthermore, the study was conducted in a single center, which may not represent the diversity of experiences across different geographic regions and different healthcare systems. Hence, the findings may be influenced by local factors, including the specific organization of public health services in Croatia, as well as regional COVID-19 incidence and response measures. Moreover, although validated instruments were used (K10, IES-R, SOC-29) for measuring psychological distress, sense of coherence, and post-traumatic stress, on the other hand, the self-reported form introduces the potential for response bias and subjective interpretation of symptoms. Additionally, while the biological sampling was standardized, it could have been affected by participant adherence to saliva collection protocols outside the laboratory setting. Hence, it was impossible to eliminate all of possible confounding effects. Lastly, the observational and correlational nature of the study precludes causal inference.

## 5. Conclusions

The findings of this study could possibly have important implications for occupational health interventions and public health workforce development. Firstly, the elevated cortisol levels and psychological distress observed during pandemic peaks highlight the need for real-time monitoring of stress biomarkers and mental health symptoms in PHWs during crisis situations. Early identification of at-risk individuals through screening programs could facilitate timely interventions, including psychological counseling, peer support, workload adjustments and referral to mental health services. Secondly, interventions targeting sense of coherence may enhance resilience and buffer against occupational stress. Organizational strategies to strengthen comprehensibility, manageability, and meaningfulness could improve mental health outcomes. Lastly, the specific vulnerabilities of epidemiologists and other PHWs engaged in pandemic response activities warrant targeted support. Contact tracers, epidemiologists, and laboratory personnel may experience secondary traumatic stress from repeated exposure to distressing situations, despite not being in direct patient care roles. Training in psychological first aid, stress management, and coping strategies, as well as fostering team cohesion and social support, may mitigate adverse mental health outcomes.

## Figures and Tables

**Table 1:** Sociodemographic and work data of the study sample and comparison between epidemiologists and non-epidemiologists.

Parameter	Study Sample N = 54	Non-Epidemiologist N = 43	Epidemiologist N = 11	*p*
Age (year)	45.5 (29.0–54.0)	43.0 (28.0–53.0)	55.0 (46.5–59.5)	0.003 *
Gender (N, %)				
Male	11 (20.4)	9 (20.9)	2 (18.2)	0.827 ^†^
Female	43 (79.6)	34 (79.1)	9 (81.8)	
Marital status (N, %)				
Single	14 (25.9)	14 (32.6)	0 (0)	0.148 ^†^
Married	35 (64.8)	25 (58.1)	10 (90.9)	
Divorced	4 (7.4)	3 (7.0)	1 (9.1)	
Widowed	1 (1.9)	1 (2.3)	0 (0)	
Education level (N, %)				
High school	19 (35.2)	19 (44.2)	0	0.004 ^†^
Bachelor’s degree	5 (9.3)	5 (11.6)	0	
Master’s degree	30 (55.6)	19 (44.2)	11 (100.0)	
Working experience (N, %)				
<5 years	12 (22.2)	12 (27.9)	0 (0)	0.016
5–10 years	7 (13.0)	7 (16.3)	0 (0)	
11–20 years	15 (27.8)	10 (23.3)	5 (45.5)	
>20 years	20 (37.0)	14 (32.6)	6 (54.5)	
Working with COVID-19 (N, %)				
<6 months	5 (9.3)	5 (11.6)	0 (0)	0.012 ^†^
6–12 months	14 (25.9)	14 (32.6)	0 (0)	
>12 months	35 (64.8)	24 (55.8)	11 (100.0)	
Smoking (N, %)	11 (20.4)	9 (20.9)	2 (18.2)	0.827 ^†^
Had COVID-19 (N, %)	27 (50.0)	18 (41.9)	9 (81.8)	0.042 ^†^
COVID-19 vaccination (N, %)				
No	25 (46.3)	23 (53.5)	2 (18.2)	0.074 ^†^
Yes, one dose	7 (13.0)	4 (9.3)	3 (27.3)	
Yes, two doses	22 (40.7)	16 (37.2)	6 (54.5)	

All data is presented as whole numbers (percentage) or medians (interquartile range). ^†^ Chi-square test. * Mann–Whitney U test.

**Table 2:** Questionnaire score and comparison between epidemiologists and non-epidemiologists.

Parameter	Study Sample N = 54	Non-Epidemiologist N = 43	Epidemiologist N = 11	*p*
SOC-29 (score)	124.1 ± 10.7	125.6 ± 10.5	117.9 ± 9.1	0.029 ^†^
K10 (score)	21.2 ± 7.6	24.9 ± 7.0	20.3 ± 8.9	0.078 ^†^
IER-R (score)	3.0 (1.6–6.2)	2.8 (1.7–6.1)	4.5 (1.6–7.6)	0.367 *
Intrusion	1.0 (0.5–2.0)	1.0 (0.3–1.9)	1.7 (0.6–2.7)	0.200 *
Avoidance	1.2 (0.7–2.1)	1.2 (0.7–2.1)	1.2 (0.5–2.0)	0.603 *
Hyperarousal	1.0 (0.3–2.1)	1.0 (0.2–1.8)	1.6 (0.7–2.9)	0.116 *

All data is presented as mean ± standard deviation or median (interquartile range). Abbreviations: SOC-29—Sense of Coherence Scale; K10—Kessler Psychological Distress Scale, IES-R—Impact of Events Scale—Revised. ^†^ Student *t*-test. * Mann–Whitney U test.

**Table 3:** Comparison of laboratory results between the resting phase and the stress phase.

Parameter	Resting Phase N = 54	Stress Phase N = 54	*p*
Cortisol (nmol/L)	391.0 (336.0–472.0)	365.0 (289.0–481.0)	0.379 *
IL-6 (pg/mL)	1.8 (1.5–2.7)	1.7 (1.5–2.9)	0.516 *
CAR	6.6 (3.4–16.1)	9–5 (3.9–21.9)	0.286 *
DCD	0.7 ± 0.5	0.8 ± 0.7	0.729 ^†^
MnInc	5.2 (0.3–12.0)	5.3 (1.6–13.1)	0.295 *

All data is presented as whole numbers (percentage) and medians (interquartile range). Abbreviations: IL-6—interleukin 6; CAR—cortisol awakening response, DCD—diurnal cortisol decline, MnInc—mean increase in cortisol levels. ^†^ paired *t*-test. * Wilcoxon test.

**Table 4:** Correlation matrix of the questionnaire scores and laboratory results.

Parameter	1. ^†^	2. ^†^	3. ^†^	4. ^†^	5. ^†^	6. ^†^	7. ^†^	8. ^†^	9. ^†^
1. SOC-29	1.000								
2. K10	−0.428 *	1.000							
3. IES-R	−0.277 *	0.536 *	1.000						
4. Δ Cortisol	−0.252	0.117	0.042	1.000					
5. Δ IL-6	0.031	0.119	0.075	0.331 *	1.000				
6. Δ CAR	0.083	0.266	0.128	−0.012	−0.095	1.000			
7. Δ DCD	0.142	−0.111	−0.018	0.049	0.012	0.004	1.000		
8. Δ AUCg	0.229	0.050	0.003	−0.054	−0.041	0.032	0.101	1.000	
9. Δ MnInc	0.284 *	−0.109	−0.266	−0.020	−0.001	−0.002	0.001	0.033	1.000

Abbreviations: SOC-29—Sense of Coherence Scale; K10—Kessler Psychological Distress Scale, IES-R—Impact of Events Scale—Revised, IL-6—interleukin 6; CAR—cortisol awakening response, DCD—diurnal cortisol decline, MnInc—mean increase in cortisol levels, AUCg—area under the curve with respect to the ground. ^†^ Spearman’s correlation coefficient. * *p* < 0.05.

**Table 5:** Multiple linear regression analysis of independent predictors for Δ cortisol level.

Parameter	Β *	SE ^†^	t	*p*
SOC-29 (score)	−2.425	1.164	−2.084	0.042
K10 (score)	0.769	1.987	0.387	0.700
IES-R (score)	−3.997	5.198	−0.769	0.445
Δ IL-6 (pg/L)	12.678	6.276	2.020	0.049
Age (year)	3.647	2.412	1.512	0.137
Working experience (year)	−34.083	24.627	−1.384	0.172

Abbreviations: SOC-29—Sense of Coherence Scale; K10—Kessler Psychological Distress Scale, IES-R—Impact of Events Scale—Revised, IL-6—interleukin 6. * unstandardized coefficient β. ^†^ standard error.

## Data Availability

The data presented in this study are available on request from the corresponding author due to both privacy and ethical reasons.
